# Ultrasensitive and Selective ZPNRs-H Sensor for Sulfur Gas Molecules Detection

**DOI:** 10.3390/nano15161273

**Published:** 2025-08-18

**Authors:** Shaolong Su, Xiaodong Lv, Jian Gong, Zhi-Qiang Fan

**Affiliations:** 1 Inner Mongolia Key Laboratory for Physics and Chemistry of Functional Materials, College of Physics and Electronic Information, Inner Mongolia Normal University, Hohhot 010022, China; 20200032@imnu.edu.cn; 2 Ordos Institute of Technology, Ordos 017000, China; 3 Hunan Provincial Key Laboratory of Flexible Electronic Materials Genome Engineering, School of Physics and Electronic Science, Changsha University of Science and Technology, Changsha 410114, China

**Keywords:** ZPNRS-H, first-principles, gas molecules, conductivity

## Abstract

The exceptional sensing properties of hydrogen-saturated zigzag phosphorene nanoribbons (ZPNRs-H) for sulfur-containing gases, namely SO_3_, SO_2_, and H_2_S, were investigated using first-principles calculations based on density functional theory. The total energy, adsorption energy, and Mulliken charge transfer were assessed to evaluate the adsorption properties of the ZPNRs-H towards these gases. Notably, the ZPNRs-H exhibits physical adsorption for SO_2_ and H_2_S gas molecules, while demonstrating chemical adsorption for SO_3_, characterized by a substantial adsorption energy and pronounced charge transfer. Furthermore, the adsorption of SO_3_ significantly modulates the electronic density of states near the Fermi level of ZPNRs-H. The current–voltage (I–V) characteristics unveil a remarkable enhancement in conductivity post-SO_3_ adsorption, underscoring the high sensitivity of ZPNRs-H towards SO_3_. Our findings provide profound theoretical insights, heralding the potential of ZPNRs-H as a cutting-edge sensor for SO_3_ detection.

## 1. Introduction

With escalating environmental challenges, environmental monitoring has emerged as a pivotal tool in environmental governance [1,2]. Real-time monitoring of air pollutants is crucial for safeguarding human health against the adverse effects of harmful gases [3]. Sulfur dioxide (SO_2_) and sulfur trioxide (SO_3_) are highly corrosive and irritant gases, significantly contributing to atmospheric pollution and acid rain formation [4]. Hydrogen sulfide (H_2_S), another prevalent toxic air pollutant, poses severe health risks. Exposure to sulfur-containing gases (SO_2_, SO_3_, and H_2_S) may escalate the risk of respiratory and lung diseases, as well as certain cancers [5]. These gases also do great harm to organisms and the environment. Therefore, the monitoring of sulfur gas is very important, and the gas sensor for sulfur gas has also begun to be developed [6,7]. However, solid electrolytes or metal oxides (SnO_2_, FeO_2_) sensors [8,9,10] have various limitations, such as the low sensitivity limits and high operating temperatures. Innovative approaches to enhancing device performance involve leveraging emerging nanomaterials.

Two-dimensional (2D) materials have drawn considerable attention owing to their special physical, chemical, and electronic properties, including atomic-scale thickness, rapid carrier mobility, extreme sensitivity, ease of device fabrication, and rapid room-temperature operation [11,12,13,14,15,16,17]. Several 2D materials, such as graphene, MoS_2_, and SnSe, have demonstrated outstanding performances in detecting various gases [18,19,20]. Recently, few-layered black phosphorus, known as phosphorene, has been successfully synthesized [21,22]. Phosphorene exhibits superior properties compared to other 2D materials due to its puckered wave-like structure with *sp*^3^ bonding, which endows it with an extremely high surface-to-volume ratio and a lower out-of-plane electrical conductance [23,24]. These properties enable phosphorene to exhibit a more sensitive response to target gas species near its surface. Extensive research has been conducted on the adsorption properties of various gas molecules (CO, H_2_O, O_2_, NO, SO_2_, NH_3_, NO_2_, CO_2_, H_2_S, and H_2_) on 2D few-layered phosphorene [25,26,27,28,29]. However, phosphorene demonstrates limited sensitivity and selectivity towards sulfur-containing gases, particularly SO_2_ and H_2_S [30]. Efforts have been made to enhance the adsorption sensitivity of SO_2_ and H_2_S on phosphorene nanosheets by introducing defects and impurities [31]. More recently, the electronic and adsorption properties of NO_2_ and SO_3_ gas molecules on hydrogenated armchair phosphorene nanoribbons (APNRs) and zigzag phosphorene nanoribbons (ZPNRs) were investigated, respectively [27,32]. However, prior to this study, no work had reported on the sensitivity and selectivity of sulfur-containing gases (SO_2_, SO_3_, and H_2_S) on hydrogenated zigzag phosphorene nanoribbons (ZPNRs-H). Therefore, we systematically investigated the electronic structures, sensing performances, and transport properties of sulfur-containing gases adsorbed on the ZPNRs-H using first principles. Our results indicate that the adsorption energy and charge transfer of the ZPNRs-H for SO_3_ are significantly higher than for SO_2_ and H_2_S. The current–voltage (I–V) curves reveal a substantial improvement in the conductivity of ZPNRs-H upon SO_3_ adsorption, which indicate the potential of ZPNRs-H as a cutting-edge sensor for SO_3_ detection.

## 2. Computational Methods

In this study, all computations were carried out utilizing the first-principles software package Atomistix ToolKit 11.2.3 (ATK). This package is grounded in density functional theory (DFT) and is used in conjunction with the non-equilibrium Green’s function (NEGF) [33,34]. For the exchange-correlation potential, we employed the generalized gradient approximation (GGA) with the Perdew–Burke–Ernzerhof (PBE) functional. To account for the van der Waals (vdW) correction, we adopted the Grimme’s DFT-D3 dispersion correction method, which was chosen due to its relatively fast computational speed and high accuracy. For all atoms, the wave function was expanded using the double-zeta plus polarization (DZP) basis set. The density mesh cutoff energy was manually set as 300 Ry to describe the real space grid. The *k*-point samplings of 1 × 1 × 21 were applied to calculate the bulk’s electronic structures, and 1 × 1 × 150 was applied to calculate the device’s electronic transport properties. To avoid interactions between adjacent supercells, the vacuum region was determined to be at least 15 Å. The geometric structures were optimized until the residual force on each atom was reduced to less than 0.01 eV Å^−1^. When a bias voltage is applied, the current can be calculated using the Landauer formula [35]:

$$I(V_{b})=\frac{2e}{h}\int T(E,V_{b})[f_{L}(E,V_{b})-f_{R}(E,V_{b})]dE$$

where $V_{b}$ is the bias voltage, $T(E,V_{b})$ is the transmission coefficient, and $f_{L}(E,V_{b})$ and $f_{R}(E,V_{b})$ are the Fermi-Dirac distribution functions of the left and right electrodes.

## 3. Results and Discussion

**Figure 1:**
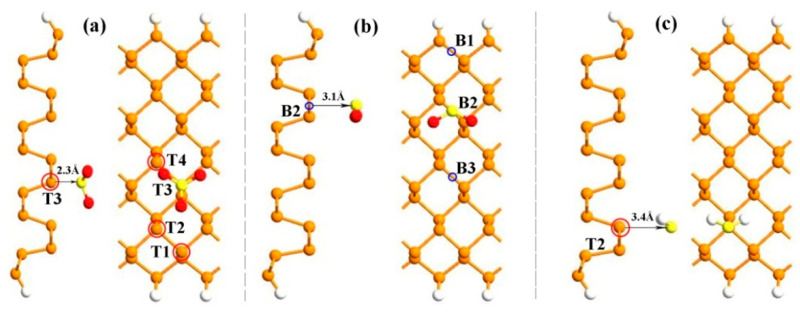
Side and top perspectives for the most stable adsorption structures of the sulfide gas molecules: (**a**) SO_3_, (**b**) SO_2_, and (**c**) H_2_S on the ZPNRs-H. The red color in its entirety represents the most stable adsorption sites. The red spheres represent oxygen (O) atoms, the yellow spheres represent sulfur (S) atoms, and the white spheres represent hydrogen (H) atoms.

The side and top perspectives of the stable configurations of different gas molecules adsorbed on ZPNR-H are depicted in Figure 1. We employ a two unit-cell structure of the ZPNRs-H as the adsorption substrate. The optimized lattice parameters are determined to be *a* = 4.61 Å and *b* = 3.32 Å, which are consistent with previous studies [36]. To determine the adsorption orientation and position of gas molecules on the surface of ZPNRs-H, the total energy was systemically investigated. When a single SO_3_ molecule is adsorbed on ZPNRs-H, it aligns along the adsorption direction, and its sulfur atoms are near the adsorption substrate. Figure 1a indicates four points of the top site of phosphorus atom (T1, T2, T3, T4), and the most stable distance between S atom and point T3 is 2.3 Å. When a single SO_2_ gas molecule is adsorbed on the ZPNRs-H, the SO_2_ molecule is parallel to the ZPNRs-H. Figure 1b indicates three points of the bridge site of the ZPNRs-H surface (B1, B2, B3), and the most stable adsorption distance between SO_2_ and site B2 is 3.1 Å. Figure 1c illustrates a H_2_S gas molecule adsorbed on the T2 position of the phosphorus atom. The most stable distance between H_2_S and site T2 is 3.4 Å.

**Figure 2:**
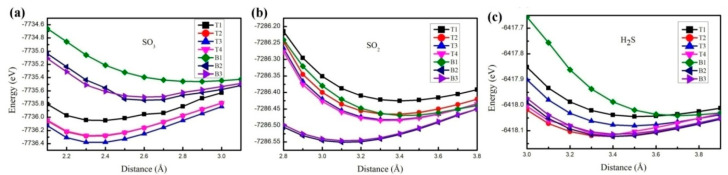
Total energies change with the different adsorption distances for (**a**) SO_3_, (**b**) SO_2_, and (**c**) H_2_S on the ZPNRs-H, respectively. T1, T2, T3, and T4 indicate the different adsorption sites on the top, and B1, B2, and B3 indicate the different adsorption sites on the bridge, respectively.

As depicted in Figure 2, to identify a stable adsorption structure, we further explored the total energy of adsorption systems with various adsorption distances. Through determining the lowest total energy, we can ascertain a stable adsorption distance. Figure 2a presents the variations in total energy for different adsorption distances of SO_3_ on ZPNRs-H. When SO_3_ is adsorbed at top-site T1, the stable adsorption distance is 2.4 Å. For adsorption at top-sites T2, T3, and T4, the stable adsorption distance is 2.3 Å. When SO_3_ is adsorbed at bridge-sites B2 and B3, the stable adsorption distance is 2.6 Å. However, the stable adsorption distance rises to 2.9 Å when SO_3_ is adsorbed at bridge-site B1. Additionally, with top-site T3, the system exhibits the lowest energy, and the stable adsorption distance is 2.3 Å. For the SO_2_ gas molecule, the system attains the lowest energy when it is adsorbed at bridge-site B2, with a stable adsorption distance of 3.1 Å. For the H_2_S gas molecule, the system reaches the lowest energy when it is adsorbed at top-site T2, and the stable adsorption distance is 3.4 Å. Among these three molecules, the stable adsorption distance between SO_3_ and the ZPNRs-H surface for the system with the lowest energy is the shortest.

**Figure 3:**
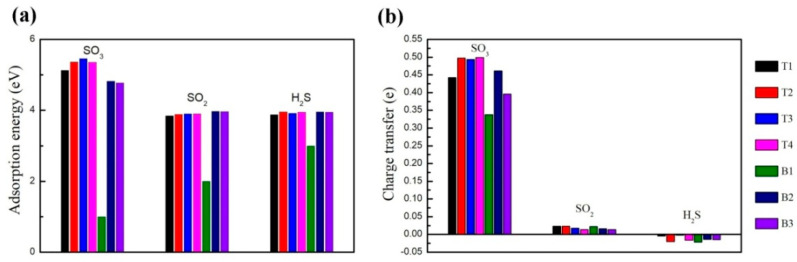
(**a**) Adsorption energies and (**b**) charge transfers for SO_3_, SO_2_, and H_2_S, on the different top and bridge sites, respectively.

To investigate the adsorption stability of various gas molecules on the ZPNRs-H, the charge transfer and adsorption energy of SO_3_, SO_2_, and H_2_S on the sites of T1, T2, T3, T4, B1, B2, and B3 were calculated, respectively, as shown in Figure 3, which is defined through the following formula:

*E*_a_ = *E*_ZPNR_ + *E*_gas_ − *E*_combined_,

where *E*_ZPNR_ and *E*_gas_ are the energies of the isolated ZPNR and the isolated molecule, respectively [37,38]. *E*_combined_ refers to the total energies of system after the ZPNR adsorbed gas molecules. The larger the absolute value of the *E*_a_, the stronger the adsorption of gas molecules on the ZPNRs-H. As shown in Figure 3a, the adsorption energy of SO_3_ on ZPNRs-H is higher than that of other molecules, except for adsorption on the B1 bridge site, which indicates a higher level sensitivity for SO_3_ adsorption on the ZPNRs-H. The adsorption energy of SO_3_ on ZPNRs-H reaches its maximum when SO_3_ is adsorbed at the T3 top site, indicating that it is relatively stable at this site. In contrast, the adsorption energy of SO_2_ on ZPNRs-H is the highest when adsorbed at the B2 bridge site. The adsorption energy of H_2_S on ZPNRs-H is the highest at the T2 top site, indicating its relative stability there. Compared to the T2, T3, and T4 positions, the adsorption energy of SO_3_, SO_2_, and H_2_S at the T1 site is the lowest. Additionally, compared to the B2 and B3 positions, the adsorption energy of SO_3_, SO_2_, and H_2_S at the B1 site is the lowest, which implies that the boundary effect makes it difficult for gas molecules adsorbed at the edge of ZPNRs-H. Charge transfer is another crucial factor for estimating the interaction between gas molecules and host materials [39,40]. As shown in Figure 3b, the charge transfer of SO_3_ adsorbed on the ZPNRs-H is the most significant, which explains why the adsorption energies of SO_3_ on ZPNRs-H are the highest. The charge transfer of SO_3_, SO_2_, and H_2_S on the sites T3, B2, and T2 is larger than the other sites, respectively. The positive and negative values indicate the ZPNR transfer and extract charge from these gas molecules, respectively.

**Figure 4:**
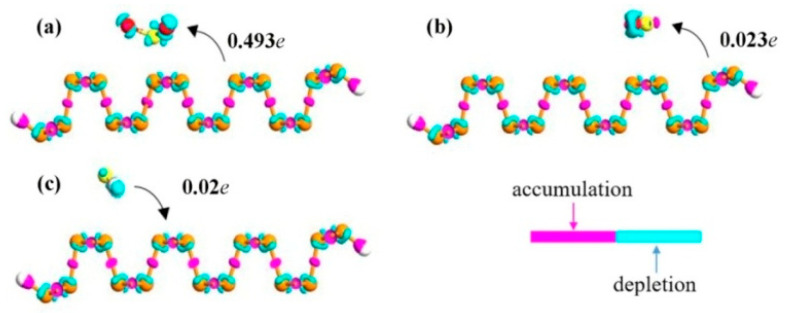
Side views of the electron difference densities (EDD) calculation for (**a**) SO_3_, (**b**) SO_2_, and (**c**) H_2_S adsorbed on the ZPNRs-H. The iso-value is 0.2 au. The cyan and purple regions indicate electron depletion and accumulation, respectively. The direction of charge transfer is shown by the arrow.

To further explore the properties of charge transfer between gas molecules and the ZPNRs-H, the electron difference densities (EDD) were investigated as shown in Figure 4, where the cyan and purple regions indicate electron depletion and accumulation, respectively. Our analysis of the Mulliken charge transfer results reveals that, for SO_3_ and SO_2_ adsorption on the T3 and B2 sites of the ZPNRs-H, respectively, there is an accumulation in charge on the gas molecules and a depletion in charge on the ZPNRs-H surface. Conversely, for H_2_S adsorption on the T2 site of ZPNRs-H, we observe a depletion in charge on the gas molecules and an accumulation in charge on the ZPNRs-H surface. The results reveal that SO_3_ and SO_2_ act as charge acceptors, acquiring 0.49 e^−^ and 0.02 e^−^ from the ZPNRs-H surface, respectively. Conversely, H_2_S acts as a charge donor, providing 0.02 e^−^ to the ZPNR. Notably, the charge transfer from the ZPNRs-H to the SO_3_ is significantly greater than that for the SO_2_ and H_2_S molecules. Furthermore, we found that the charge transfers are much more significant in the case of the SO_3_ molecule on ZPNR, which indicate that ZPNRs-H is more suitable for a SO_3_ molecular sensor as shown in Figure 4.

**Figure 5:**
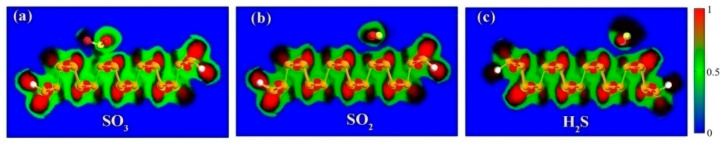
Side views of the electron localization function (ELF) calculation for (**a**) SO_3_, (**b**) SO_2_, and (**c**) H_2_S adsorbed on the ZPNRs-H. The iso-surface value is 0.002 e/Å^3^.

The charge distribution of the adsorption system was further investigated by calculating the electron localization function (ELF), as shown in Figure 5. In the case of the SO_3_ molecule adsorbed on the ZPNRs-H, there was a slight overlap between the molecule and the ZPNR in the electron localization function, indicating a significant redistribution of surface charge on the ZPNR. This finding is consistent with the conclusions drawn from the Mulliken charge transfer analysis shown in Figure 4a. Therefore, it is reasonable to consider the adsorption of SO_3_ on the ZPNRs-H as chemisorption due to the large adsorption energy, charge transfer, and slight overlap in electron localization. Hence, it can be inferred that gas sensors based on ZPNRs-H exhibit high sensitivity and selectivity towards SO_3_ molecules in sulfur-containing gases. However, the adsorption process is irreversible due to the presence of chemical bonds [41]. On the other hand, for SO_2_ and H_2_S molecules, there is no electron localization overlap between the gas molecules and the ZPNR, indicating that the ZPNRs-H is insensitive to these gas molecules.

**Figure 6:**
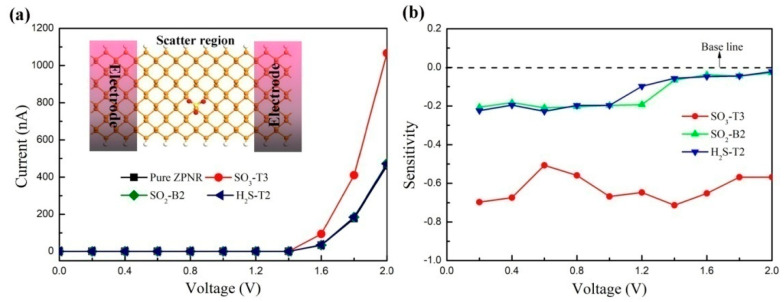
(**a**) Current–voltage (I–V) curves of the ZPNRs-H before and after SO_3_, SO_2_, and H_2_S adsorption. Inset is the top view of the two-probe system of the ZPNRs-H before and after SO_3_, SO_2_, and H_2_S adsorption. (**b**) Sensitivity of the ZPNRs-H with adsorption of different gas molecules.

To examine the variability of conductivity more effectively, the I–V response for the ZPNRs-H sensor before and after the adsorption of gas molecules was calculated, as shown in Figure 6a. We observed that there is no current flowing when the bias voltage applied to the ZPNRs-H sensor is below 1.4 V due to the 1.34 eV band gap of the ZPNRs-H. However, when the bias voltage exceeds 1.4 V, the current increases significantly with the increasing bias voltage. Notably, in the case of SO_3_ adsorption, the current is significantly higher compared to other situations as the bias voltage is increased from 1.4 V to 2.0 V. Specifically, at a bias voltage of 1.6 V, the current of the ZPNRs-H sensor is measured at 32.85 nA, while after the adsorption of SO_2_ and H_2_S, the currents are 34.13 nA and 34.48 nA, respectively. In contrast, after the adsorption of SO_3_, the current reaches 94.38 nA, indicating an increase of approximately three times compared to previous results. Similarly, at a bias voltage of 2.0 V, the current of the ZPNRs-H sensor is 460.54 nA, which increases to 473.69 nA and 470.8 nA after the adsorption of SO_2_ and H_2_S, respectively. However, with the adsorption of SO_3_, the current rises to 1067.8 nA. These increases in current suggest that the resistance of the ZPNRs-H decreases after the adsorption of SO_3_, which can be easily measured experimentally. This decrease in resistance is attributed to the greater charge transfer between the ZPNRs-H and SO_3_ molecule.

To assess the sensitivity of the ZPNRs-H sensor to different gas molecules, we calculated the sensitivity before and after the adsorption of various gas molecules, as shown in Figure 6b, using the formula:

*S*(%) = (Δ*R*/*R*) × 100%,

where Δ*R* represents the resistance change after the adsorption of the gas molecule, and *R* is the resistance of the pure ZPNRs-H. The maximum absolute values of the sensitivities are approximately 71.3%, 20.2%, and 22.8% for SO_3_, SO_2_, and H_2_S gas molecules, respectively, on the ZPNRs-H sensor. Clearly, the ZPNRs-H systems possess higher sensitivity compared with the AsP monolayer [13]. The current response of the ZPNRs-H sensor to SO_3_ is particularly pronounced, indicating high sensitivity and selectivity towards SO_3_ molecules.

**Figure 7:**
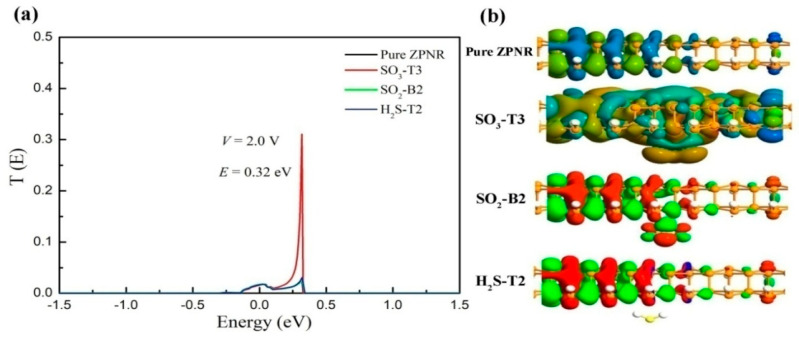
(**a**) Transmission function at 2.0 V bias of the ZPNRs-H before and after SO_3_, SO_2_, and H_2_S adsorption responsible for maximum transmission (eigenchannels at 0.32 eV). (**b**) Eigen states at 2.0 V on the transmission peaks of the ZPNRs-H before and after SO_3_, SO_2,_ and H_2_S adsorption; isosurface value is 0.06 e/Å^3^.

To gain a better understanding of why the current of the ZPNRs-H sensor is significantly larger after the adsorption of SO_3_ molecules compared to other cases, we performed calculations on the transmission function of the sensor before and after the adsorption of SO_3_, SO_2_, and H_2_S molecules at a bias voltage of 2.0 V, as shown in Figure 7a. From the figure, it can be observed that the transmission function tends to be similar after the adsorption of SO_2_ and H_2_S under a bias voltage of 2.0 V. However, the maximum transmission peak after the adsorption of SO_3_ molecules is markedly higher than in other cases, occurring at an energy of *E* is 0.32 eV, which accounts for the larger current. To examine electron transport in the scattering region more clearly, we calculated the intrinsic transport channel corresponding to the maximum transmission peak after the adsorption of SO_3_, SO_2_, and H_2_S molecules, as depicted in Figure 7b. It is evident that a significant number of electronic states are transported from the source region to the drain region in the ZPNRs-H sensor after the adsorption of SO_3_ molecules, with the SO_3_ molecule playing an important role in facilitating this electronic state transport. However, after the adsorption of SO_2_ and H_2_S molecules, numerous electronic states are scattered and fail to reach the drain region.

## 4. Conclusions

In this work, we conducted a comprehensive investigation into the interaction and electron transport characteristics of ZPNRs-H with SO_3_, SO_2_, and H_2_S gas molecules. Our aim was to determine the most stable energy configuration for the adsorption of these gases on ZPNRs-H. The results reveal that ZPNRs-H exhibits a higher sensitivity towards SO_3_ gas molecules. Specifically, both the adsorption energy and the amount of charge transfer for SO_3_ are greater than those for SO_2_ and H_2_S gas molecules. This conclusion is further supported by the electronic local function (ELF). These characteristics make ZPNRs-H a promising candidate as an excellent sensor for detecting SO_3_ gas. The current–voltage curve indicates a significant improvement in the conductivity of ZPNRs-H when SO_3_ is adsorbed. In contrast, the conductivity remains almost unchanged when SO_2_ and H_2_S are adsorbed on ZPNRs-H. This demonstrates that ZPNRs-H shows high sensitivity and selectivity towards SO_3_ gas molecules. Based on these results, we can conclude that the ZPNRs-H sensor is a potentially highly sensitive and selective sensor for detecting SO_3_ gas molecules.

## Data Availability

Data are contained within the article.

## References

[1] Yuan W., Shi G. (2013). Graphene-based gas sensors. J. Mater. Chem. A.

[2] Meng Z., Stolz R.M., Mendecki L., Mirica K.A. (2019). Electrically transduced chemical sensors based on two-dimensional nanomaterials. Chem. Rev..

[3] Shimizu Y. (2001). Improvement of SO_2_ sensing properties of WO_3_ by noble metal loading. Sens. Actuators B.

[4] Cheng Y.F., Meng R.S., Tan C.J., Chen X.P., Xiao J. (2018). Selective gas adsorption and I-V response of monolayer boron phosphide introduced by dopants: A first-principles study. Appl. Surf. Sci..

[5] Lee S.C., Hwang B.W., Lee S.J., Choi H.Y., Kim S.Y., Jung S.Y., Ragupathy D., Lee D.D., Kim J.C. (2011). A novel tin oxide-based recoverable thick film SO_2_ gas sensor promoted with magnesium and vanadium oxides. Sens. Actuator B-Chem..

[6] Kou L.Z., Frauenheim C.F., Chen T. (2014). Phosphorene as a superior gas sensor: Selective adsorption and distinct I-V Response. J. Phys. Chem. Lett..

[7] Guo S., Yuan L., Liu X., Zhou W., Song X., Zhang S. (2017). First-principles study of SO_2_ sensors based on phosphorene and its isoelectronic counterparts: GeS, GeSe, SnS, SnSe. Chem. Phys. Lett..

[8] Imanaka N., Yamaguchi Y., Adachi G., Shiokawa J. (1987). Sulfur dioxide gas detection with Na_2_SO_4_-Li_2_SO_4_-Y_2_(SO_4_)_3_-SiO_2_ solid electrolyte by a solid reference electrode method. J. Electrochem. Soc..

[9] Das S., Chakraborty S., Parkash O., Kumar D., Bandyopadhyay S., Samudrala S.K., Sen A., Maiti H.S. (2008). Vanadium doped tin dioxide as a novel sulfur dioxide sensor. Talanta.

[10] Fergus J.W. (2008). A review of electrolyte and electrode materials for high temperature electrochemical CO_2_ and SO_2_ gas sensors. Sens. Actuator B-Chem..

[11] Yashina L.V., Zyubin A.S., Püttner R., Zyubina T.S., Neudachina V.S., Stojanov P., Riley J., Dedyulin S.N., Brzhezinskaya M.M., Shtanov V.I. (2011). The Oxidation of PbS(001) surface with O_2_ and air studied with photoelectron spectroscopy and ab initio modelling. Surf. Sci..

[12] Cho S.Y., Lee Y., Koh H.J., Jung H., Kim J.S., Yoo H.W., Kim J., Jung H.T. (2016). Superior chemical sensing performance of black phosphorus: Comparison with MoS_2_ and graphene. Adv. Mater..

[13] Wang J., Yang G.F., Xue J.J., Lei J.M., Chen D.J., Lu H., Zhang R., Zheng Y.D. (2018). High Sensitivity and Selectivity of AsP Sensor in Detecting SF6 Decomposition Gases. Sci. Rep..

[14] Liu Q.Q., Li J.J., Wu D., Deng X.Q., Zhang Z.H., Fan Z.Q., Chen K.Q. (2021). Gate-controlled reversible rectifying behavior investigated in a two-dimensional MoS_2_ diode. Phys. Rev. B.

[15] Rabchinskii M.K., Sysoev V.V., Ryzhkov S.A., Eliseyev I.A., Stolyarova D.Y., Antonov G.A., Struchkov N.S., Brzhezinskaya M., Kirilenko D.A., Pavlov S.I. (2022). A blueprint for the synthesis and characterization of thiolated Graphene. Nanomaterials.

[16] Yang J., Sun R.S., Bao X., Liu J., Ng J.W., Tang B., Liu Z. (2025). Enhancing selectivity of two-dimensional materials-based gas sensors. Adv. Funct. Mater..

[17] Kadam S.A. (2025). Advancements in monolayer TMD-based gas sensors: Synthesis, mechanisms, elec tronic structure engineering, and flexible wearable sensors for real-world applications and future prospects. Chem. Eng. J..

[18] Schedin F., Geim A.K., Morozov S.V., Hill E.W., Blake P., Katsnelson M.I., Novoselov K.S. (2007). Detection of individual gas molecules adsorbed on graphene. Nat. Mater..

[19] Friedman A.L., Perkins F.K., Cobas E., Jernigan G.G., Campbell P.M., Hanbicki A.T., Jonker B.T. (2014). Chemical vapor sensing of two-dimensional MoS_2_ field effect transistor devices. Solid-State Electron.

[20] Salih E., Ayesh A.I. (2021). Pt-doped armchair graphene nanoribbon as a promising gas sensor for CO and CO_2_: DFT study. Physica E.

[21] Castellanos-Gomez A. (2015). Black phosphorus: Narrow gap, wide applications. J. Phys. Chem. Lett..

[22] Li L.K., Yang F.Y., Ye G.J., Zhang Z.C., Zhu Z.W., Lou W.K., Zhou X.Y., Li L., Watanabe K., Taniguchi T. (2016). Quantum Hall effect in black phosphorus two-dimensional electron system. Nat. Nanotechnol..

[23] Xia F.N., Wang H., Jia Y.C. (2014). Rediscovering black phosphorus as an anisotropic layered material for optoelectronics and electronics. Nat. Commun..

[24] Ray S.J. (2016). First-principles study of MoS_2_, phosphorene and graphene based single electron transistor for gas sensing applications. Sens. Actuator B-Chem..

[25] Khan M.S., Srivastava A., Pandey R. (2016). Electronic properties of a pristine and NH_3_/NO_2_ adsorbed buckled arsenene monolayer. RSC Adv..

[26] Kuang A., Kuang M., Yuan H., Wang G., Chen H., Yang X. (2017). Acidic gases (CO_2_, NO_2_ and SO_2_) cap ture and dissociation on metal decorated phosphorene. Appl. Surf. Sci..

[27] Nagarajan V., Chandiramouli R. (2017). Adsorption of NO_2_ molecules on armchair phosphorene nanosheet for nano sensor applications–A first-principles study. J. Mol. Graphics Modell..

[28] Huang C.S., Murat A., Babar V., Montes E., Schwingenschlögl U. (2018). Adsorption of the gas molecules NH_3_, NO, NO_2_, and CO on borophene. J. Phys. Chem. C.

[29] Lei S.Y., Yu Z.Y., Shen H.Y., Sun X.L., Wan N., Yu H. (2018). CO adsorption on metal-decorated phos phorene. ACS Omega.

[30] Wang Y., Lei S., Gao R., Sun X., Chen J. (2021). Effect of metal decoration on sulfur-based gas molecules adsorption on phosphorene. Sci. Rep..

[31] Kaewmaraya T., Ngarnwongwan L., Moontragoon P., Karton A., Hussain T. (2018). Drastic improvement in gas-sensing characteristics of phosphorene nanosheets under vacancy defects and elemental functionalization. J. Phys. Chem. C.

[32] Su S.L., Gong J., Fan Z.Q. (2020). Selective adsorption of harmful molecules on zigzag phosphorene nano ribbon for sensing applications. Physica E.

[33] Taylor J., Guo H., Wang J. (2001). Ab initio modeling of open systems: Charge transfer, electron conduction, and molecular switching of a C-60 device. Phys. Rev. B.

[34] Brandbyge M., Mozos J.L., Ordejon P., Taylor J., Stokbro K. (2002). Density-functional method for nonequilibrium electron transport. Phys. Rev. B.

[35] Büttiker M., Imry Y., Landauer R., Pinhas S. (1985). Generalized many-channel conductance formula with application to small rings. Phys. Rev. B.

[36] Jin J.C., Wang Z.Y., Dai X.Q., Xiao J.R., Long M.Q., Chen T. (2019). The electronic and transport properties of the phosphorene nanoribbons. Mater. Res. Express.

[37] Nagarajan V., Bhuvaneswari R., Chandiramouli R. (2024). Phosphoborane nanosheets as a sensing element for liquefied petroleum gas a first-principles study. Chem. Phys. Lett..

[38] Hussain T., Kaewmaraya T., Chakraborty S., Vovusha H., Amornkitbamrung V., Ahuja R. (2018). Defected and functionalized germanene-based nanosensors under sulfur comprising gas exposure. ACS Sens..

[39] Li H., Liu Z., Liu G., Yang N., Wu Q., Xiao X., Chen T. (2024). Biaxila strain modulated high anisotropic gas-sensing performance of C_5_N-based two-dimensional devices: A first-principles study. Surf. Interfaces.

[40] He H., Hao Z.W., Lu X.Q., Dong M.M., Li Z.L., Wang C.K., Fu X.X. (2024). Black phosphorene with Au modification: Oxynitride remover and hydrogen sensor. Appl. Surf. Sci..

[41] Ye H.Y., Liu L., Xu Y.X., Wang L.Y., Chen X.P., Zhang K., Liu Y.F., Koh S.W., Zhang G.Q. (2019). SnSe monolayer: A promising candidate of SO_2_ sensor with high adsorption quantity. Appl. Surf. Sci..

